# Burden and trends of hospitalisations associated with pulmonary non-tuberculous mycobacterial infections in Germany, 2005–2011

**DOI:** 10.1186/1471-2334-13-231

**Published:** 2013-05-21

**Authors:** Felix C Ringshausen, Rosa-Marie Apel, Franz-Christoph Bange, Andrés de Roux, Mathias W Pletz, Jessica Rademacher, Hendrik Suhling, Dirk Wagner, Tobias Welte

**Affiliations:** 1 Department of Respiratory Medicine, Hannover Medical School, Hannover, Germany; 2 Institute for Medical Microbiology and Hospital Epidemiology, Hannover Medical School, Hannover, Germany; 3 Centre for Respiratory Medicine at the Charlottenburg Castle, Berlin, Germany; 4 Centre for Infectious Diseases and Infection Control, Jena University Hospital, Jena, Germany; 5 Centre for Infectious Diseases and Travel Medicine and Centre for Chronic Immunodeficiency, University Hospital Freiburg, Freiburg, Germany

**Keywords:** Atypical mycobacterial infection, Bronchiectasis, Clinical epidemiology, COPD epidemiology, Cystic fibrosis, Health services research, International classification of diseases, *Mycobacterium avium* complex, Non-tuberculous mycobacteria, Tuberculosis

## Abstract

**Background:**

Representative population-based data on the epidemiology of pulmonary non-tuberculous mycobacterial (PNTM) infections in Europe are limited. However, these data are needed in order to optimise patient care and to facilitate the allocation of healthcare resources. The aim of the present study was to investigate the current burden and the trends of PNTM infection-associated hospitalisations in Germany.

**Methods:**

*International Classification of Diseases, 10th revision* (ICD-10) discharge diagnosis codes were extracted from the official nationwide diagnosis-related groups (DRG) hospital statistics in order to identify PNTM infection-associated hospitalisations (ICD-10 code A31.0) between 2005 and 2011. Poisson log-linear regression analysis was used to assess the significance of trends.

**Results:**

Overall, 5,959 records with PNTM infection as any hospital discharge diagnosis were extracted from more than 125 million hospitalisations. The average annual age-adjusted rate was 0.91 hospitalisations per 100,000 population. Hospitalisation rates increased during the study period for both males and females, with the highest rate of 3.0 hospitalisations per 100,000 population among elderly men, but the most pronounced average increase of 6.4%/year among females, particularly those of young and middle age, and hospitalisations associated with cystic fibrosis. Overall, chronic obstructive pulmonary disease (COPD) was the most frequent PNTM infection-associated condition in 28.9% of hospitalisations and also showed a significant average annual increase of 4.8%.

**Conclusions:**

The prevalence of PNTM infection-associated hospitalisations is steadily increasing in Germany. COPD is currently the most important associated condition. Our population-based study provides evidence of a changing epidemiology of PNTM infections and highlights emerging clinical implications.

## Background

Non-tuberculous mycobacteria (NTM) are ubiquitous environmental and biologically diverse micro-organisms, some of which are associated with chronic and progressive pulmonary infections in susceptible individuals [1,2]. In general, *Mycobacterium avium* complex (MAC) is considered the most common causative agent of pulmonary NTM (PNTM) infection worldwide [3-6].

Recent epidemiologic data suggest that both the incidence and the prevalence of NTM infections are increasing worldwide [5,7-12]. Furthermore, there is growing evidence that the epidemiology of PNTM infections is changing, with immunocompetent subjects suffering from pre-existing structural lung disease and adults aged ≥50 years with a substantial proportion of never-smoking females without previous lung disease being increasingly reported from outside Europe [5,7,13,14]. Most of these studies were either solely laboratory-based or used the frequency of NTM isolates from clinical specimens in order to determine prevalence and to reason the significance of NTM infection in conjunction with clinical and radiological data according to the American Thoracic Society (ATS) and Infectious Diseases Society of America (IDSA) statement on the diagnosis of NTM disease [3,7,10,11,13-15].

However, while representative population-based data for Europe are limited [11,15], the epidemiology of PNTM infections in Germany is largely unknown. In Germany, population-based data regarding hospitalisations are available at a federal level. Although primarily used for the billing of health services, some important epidemiological evidence has originated from the analysis of *International Classification of Diseases* hospital discharge diagnosis codes and health insurance claims [9,16].

Epidemiological and health services research on PNTM infections is required in order to optimise patient care and to inform health policy. The aim of the present study was to provide an overview of the current burden and show the trends of PNTM infection-associated hospitalisations in Germany.

## Methods

### Data sources

The present study used data from the official nationwide diagnosis-related groups (DRG) hospital statistics, which are publicly accessible and provided by the Federal Statistical Office together with the Federal Health Monitoring Information System [17,18]. We extracted records for which the four-digit *International Classification of Diseases, 10th revision* (ICD-10) code A31.0, pulmonary mycobacterial infection (by definition including infection due to *Mycobacterium avium*, *M. intracellulare* and *M. kansasii*) within the three-digit category A31 (infection due to mycobacteria other than *M. tuberculosis* complex and *M. leprae*) was listed as any hospital discharge diagnosis.

All German hospitals using DRG billing of medical services are legally obligated to transmit this data in response to an annual written survey. Consequently, hospitals for prevention, rehabilitation, mental and mood disorders as well as day care units were not included in our analysis. ICD-10 was introduced in Germany in 2000. Since 2005, all associated secondary ICD-10 hospital discharge codes are additionally transmitted to the Federal Statistical Office. In general, primary ICD-10 diagnosis codes are regarded as the principal condition identified during hospitalisation, while secondary codes indicate associated or contributing conditions (comorbidities and/or complications). De-identified DRG diagnosis data were provided for the whole of Germany as absolute numbers stratified by age groups, sex and year of diagnosis. Additional variables included associated primary and secondary conditions as indicated by four-digit ICD-10 codes, overall length of hospital stay (LOS) as well as nationwide and in-hospital mortality data regarding PNTM infection as the primary diagnosis. For comparison, data regarding the overall LOS were analysed for all hospitalisations regardless of the primary ICD-10 code as well as ICD-10 category J44 (other chronic obstructive pulmonary disease, COPD). Data on PNTM infection as a cause of death are based on the official causes of death statistics from the Federal Statistical Office [18]. The data are acquired within an annual census from mandatory death certificates and statistical bulletins of mortality using the ICD-10. However, as in many other countries, NTM disease is not a notifiable condition in Germany.

### Statistical analysis

Analysis comprised all hospitalisations with PNTM infection as either a primary or a secondary hospital discharge diagnosis from 2005 through 2011. Official German census age- and sex-specific population data were used as the denominator for all calculations [18]. Age adjustment was performed by the direct method in order to control for different age distributions across Germany and to allow for comparison between different years. Age-adjusted hospitalisation rates were estimated using the latest available German Census Standard Population as the reference population. The significance of trends was calculated by Poisson log-linear regression analysis. Standard errors were scaled using Pearson’s chi-square statistics in order to account for overdispersion. Wald statistics and bootstrapping were used to estimate *p* values and 95% confidence intervals (CI), respectively. Continuous data were checked for normal distribution using the Kolmogorov-Smirnov test before calculating means. Statistical significance was set to *p*<0.05. Accordingly, differences were considered statistically significant if 95% CIs were not overlapping. Moreover, the annual percentage change (APC) of PNTM infection-associated hospitalisations was calculated. Associated diagnoses were analysed and the APC of the rate of associated primary and secondary diagnoses per 1000 hospitalisations with any diagnosis of PNTM infection was estimated. Data analysis was performed using IBM SPSS Statistics, version 20 (IBM Corp., New York, NY).

### Study approval and funding

Institutional review board approval and patient consent were not required as this study is a secondary analysis of an anonymous and publicly accessible database. There was no external funding for this study.

## Results

### Population, hospitals and hospitalisations

From 2005 to 2011, the average annual German population was 82.1 million, ranging from 82.4 million in 2005 to 81.1 million in 2011. During this period, on average 1,673 (95% CI 1,633–1,719) hospitals were subject to DRG billing of medical services, with a steady decline from 1,770 to 1,601 hospitals. The average overall number of hospitals in Germany was 2,087 (95% CI 2,067–2,108). Hence, 80% of all hospitals were included in our analysis. On average, there were 17.9 (95% CI 17.4–18.3) million hospitalisations per year, with a steady increase from 17.0 million to 18.8 million.

### Burden of PNTM infection as a hospital discharge diagnosis

**Figure 1:**
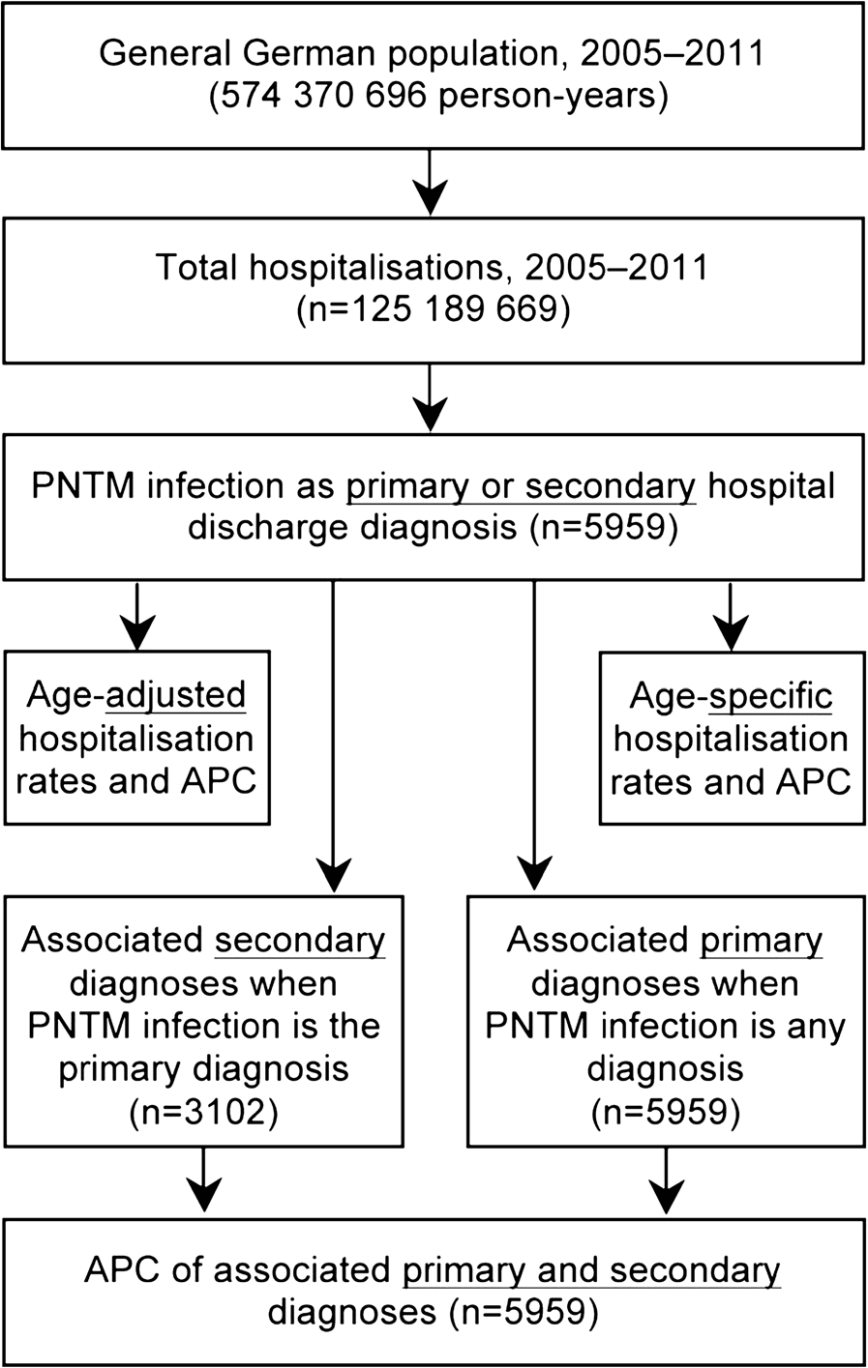
**Data flow and data analysis diagram.** APC = annual percentage change; PNTM = pulmonary non-tuberculous mycobacterial.

**Figure 2:**
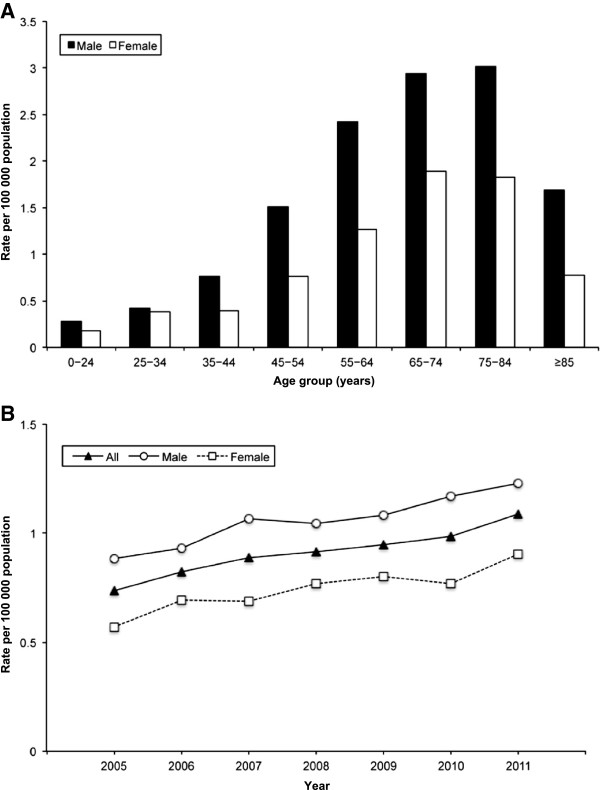
**Age-specific and age-adjusted hospitalisation rate.** (**A**) Average annual age-specific hospitalisation rate and (**B**) annual age-adjusted hospitalisation rate of pulmonary non-tuberculous mycobacterial infection as any hospital discharge diagnoses, by age group, sex and year.

From 2005 to 2011, a total of 125.2 million hospitalisations were analysed over an observational period of 574.4 million person-years. Overall, 3,102 hospitalisations with PNTM infections as the primary hospital discharge diagnosis and 5,959 hospitalisations as either a primary or a secondary diagnosis were identified (Figure 1). Of those, 3,607 hospitalisations (61%) were among males and 3,818 (64%) were among subjects aged ≥55 years. The overall average annual age-adjusted hospitalisation rate was 0.91 (95% CI 0.83–0.99) per 100,000 population. This rate was significantly higher among males (1.06; 95% CI 0.97–1.14) compared to females (0.74; 95% CI 0.67–0.82). However, there was considerable variation with age, with the highest age-specific hospitalisation rate of 3.0/100,000 population among elderly men (Figure 2).

### Trends of PNTM infections as a hospital discharge diagnosis

The annual number of all PNTM infection-associated hospitalisations ranged from 665 in 2005 to 1,039 in 2011. Accordingly, the annual proportion of PNTM infection-associated hospitalisations among all hospitalisations steadily increased from 0.0039% to 0.0055% during this period, with an average annual increase of 4.9% (95% CI 3.6–6.2; *p*<0.00001). The overall age-adjusted rate of PNTM infection-associated hospitalisations increased significantly from 0.73 to 1.09 per 100,000 population, with an average increase of 5.9% (95% CI 4.7–7.0; *p*<0.00001) per year. Among males, the annual age-adjusted prevalence increased from 0.88 to 1.23 per 100,000 population. This upward trend was even more pronounced among females, with an increase of 0.57 to 0.91 per 100,000 population (Table 1; Figure 2).

**Table 1:** Average annual percentage change of pulmonary non-tuberculous mycobacterial infection-associated hospitalisations, stratified by sex and age group

	**Males**			**Females**		
**Age group (years)**	**Annual % change**	**95% CI (Wald)**	***p*Value**	**Annual % change**	**95% CI (Wald)**	***p*Value**
0–24*	11.2	−1.5 to 24.0	0.085	13.7	5.9 to 22.1	0.0004
25–34*	−0.3	−5.3 to 4.7	0.90	−8.3	−20.3 to 3.8	0.18
35–44*	−0.5	−11.6 to 10.6	0.93	16.7	8.3 to 25.8	0.00005
45–54*	2.6	−1.2 to 6.5	0.18	2.3	−4.7 to 9.2	0.53
55–64*	5.7	3.2 to 8.3	<0.00001	8.9	4.5 to 13.4	<0.00001
65–74*	9.8	5.5 to 14.2	<0.00001	9.9	3.0 to 17.2	0.004
75–84*	6.9	2.9 to 11.1	0.0007	3.8	−3.2 to 10.9	0.29
≥85*	4.5	−12.3 to 21.3	0.60	−8.8	−18.4 to 0.8	0.072
All ages^#^	5.3	4.0 to 6.6	<0.00001	6.4	4.1 to 8.7	<0.00001

*Referring to the age-specific hospitalisation rate per 100,000 population. ^#^Referring to the age-adjusted hospitalisation rate per 100,000 population.

### Burden and trends of associated diagnoses

Secondary ICD-10 codes were available for analysis from all hospitalisations with a primary diagnosis of PNTM infection. Table 2 lists conditions with frequencies >1% which are commonly considered associated with and/or predisposing to PNTM infections, while Table 3 shows the ten most frequent comorbidities considered unrelated to PNTM infections.

**Table 2:** Conditions commonly associated with and/or predisposing to pulmonary non-tuberculous mycobacterial (PNTM) infection among secondary diagnoses when PNTM infection is the primary hospital discharge diagnosis (n=3,102)

**ICD-10**	**Secondary diagnosis**	**Frequency**	**Percentage**
J40–J47	Chronic lower respiratory diseases	1,671	53.9
J43, J44	COPD and emphysema	1,297	41.8
J96	Respiratory failure	633	20.4
Z29.0	Isolation care	533	17.2
R64	Cachexia	341	11.0
F17	Nicotine dependence	334	10.8
J09–J18	Influenza and pneumonia	300	9.7
J47	Bronchiectasis	199	6.4
B37	Candidiasis	189	6.1
B20–B24	Human immunodeficiency virus	172	5.5
B96.2	*Escherichia coli* as a causative agent	150	4.8
R04.2	Haemoptysis	135	4.4
A15–A19	Tuberculosis	125	4.0
F10	Alcohol-related disorders	116	3.7
I27	Pulmonary heart diseases	116	3.7
D38	Airway or chest neoplasm of uncertain or unknown behaviour	114	3.7
Z90.2	Acquired absence of (part of) lung	114	3.7
J84	Interstitial lung disease	112	3.6
U82.2	Resistance to one or more first-line antimycobacterial drugs	86	2.8
K21	Gastroesophageal reflux disease	85	2.7
J20–J22	Acute bronchitis and bronchiolitis	81	2.6
B90	Sequelae of tuberculosis	80	2.6
J90–J91	Pleural effusion	78	2.5
J60–J65	Pneumoconioses	72	2.3
M05–M06	Rheumatoid arthritis	70	2.3
B96.5	*Pseudomonas aeruginosa* as a causative agent	68	2.2
K50–K52	Inflammatory bowel diseases	67	2.2
B44	Aspergillosis	56	1.8
Z94	Transplanted organ or tissue status	54	1.7
B95.6	*Staphylococcus aureus* as a causative agent	51	1.6
A31.1–A31.9	Other non-tuberculous mycobacterial infections	49	1.6
C34	Lung cancer	49	1.6
D80–D84	Primary immunodeficiencies	41	1.3

COPD = chronic obstructive pulmonary disease; ICD-10 = *International Classification of Diseases, 10th revision*.

**Table 3:** Most frequent comorbidities unrelated to pulmonary non-tuberculous mycobacterial (PNTM) infections among secondary diagnoses when PNTM infection is the primary hospital diagnosis (n=3,102)

**ICD-10**	**Secondary diagnosis**	**Frequency**	**Percentage**
I10	Primary hypertension	744	24.0
E87	Disorders of water, electrolyte and acid–base balance	324	10.4
I25	Coronary artery disease	304	9.8
E11	Diabetes mellitus type 2	223	7.2
I11	Hypertensive heart disease	223	7.2
I48	Atrial flutter or fibrillation	201	6.5
I50	Congestive heart failure	198	6.4
M80–M81	Osteoporosis with or without pathological fracture	169	5.4
N18	Chronic kidney disease	165	5.3
E03	Hypothyroidism	148	4.8

ICD-10 = *International Classification of Diseases, 10th revision*.

**Figure 3:**
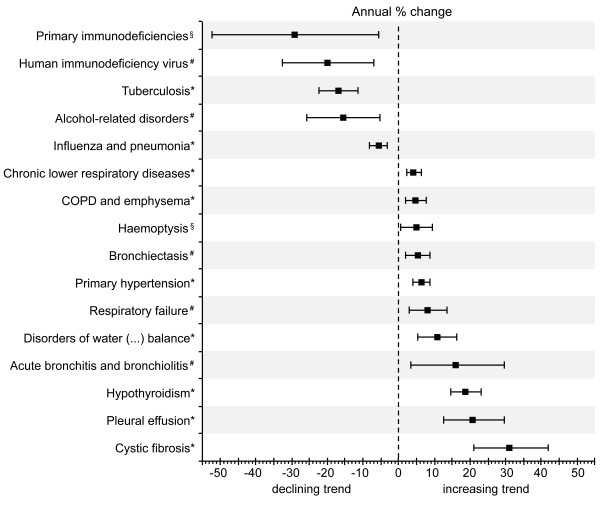
**Average annual percentage change of the rate of associated primary and secondary diagnoses per 1000 hospitalised patients with any diagnosis of pulmonary non-tuberculous mycobacterial infection.** Bars indicate 95% confidence intervals calculated from Poisson log-linear regression (Wald statistics). Non-significant trends between 2005 and 2011 are not shown. *Statistical significance at *p*<0.001. ^#^Statistical significance at *p*<0.01. ^§^Statistical significance at *p*<0.05.

In addition, this permitted the analysis of associated primary diagnoses when PNTM infection was the secondary diagnosis (Table 4) as well as the analysis of the APC of the overall rate of associated primary and secondary diagnoses (Figure 3).

**Table 4:** Primary diagnoses among hospitalisations with pulmonary non-tuberculous mycobacterial infection as any diagnosis

**ICD-10**	**Primary diagnosis**	**Frequency**	**Percentage**
A31.0	Pulmonary non-tuberculous mycobacterial infection	3,102	52.1
J40–J47	Chronic lower respiratory diseases	482	8.1
J43, J44	COPD and emphysema	423	7.1
J09–J18	Influenza and pneumonia	234	3.9
C34	Lung cancer	206	3.5
A15–A19	Tuberculosis	133	2.2
E84	Cystic Fibrosis	125	2.1
B20–B24	Human immunodeficiency virus	84	1.4
J96	Respiratory failure	71	1.2
	Other primary diagnoses <1% of hospitalisations	1,099	18.4
Total		5,959	100

COPD = chronic obstructive pulmonary disease; ICD-10 = *International Classification of Diseases, 10th revision*.

The average number of secondary diagnoses per primary diagnosis of PNTM infection was 5.5 (95% CI 5.2–5.7), with an average increase of 2.9%/year (95% CI 1.7–4.1; *p*<0.00001), ranging from 5.0 in 2005 to 6.2 in 2011. Chronic lower respiratory diseases (ICD-10 codes J40–J47) and, in particular, COPD and emphysema (ICD-10 codes J43 and J44, analysed as one entity) were the predominant secondary diagnosis of hospitalisations with PNTM infection as the primary diagnosis, respectively (Table 2). Among all records with PNTM infection as any diagnosis, PNTM infection itself was the most frequent primary diagnosis, followed by chronic lower respiratory diseases, COPD and emphysema and a variety of other primary diagnoses (Table 4).

Accordingly, chronic lower respiratory diseases as well as COPD and emphysema had by far the highest overall rates of 361 and 289 associated diagnoses per 1,000 hospitalisations with any diagnosis of PNTM infection, respectively. Figure 3 shows the downward and upward trends of the rates of PNTM infection-associated diagnoses with significant average annual changes.

### Length of hospitalisation and mortality

The mean overall LOS per hospitalisation with PNTM infection as the primary diagnosis of 18.3 (95% CI 16.8–20.0) days was significantly longer than the LOS for COPD as a primary diagnosis (ICD-10 category J44; 10.4 (95% CI 10.0–10.7) days and for all hospitalisations (all ICD-10 codes; mean 8.1 (95% CI 7.9–8.4) days). Between 2005 and 2011, the LOS showed a significant downward trend for PNTM infections (−5.0%; 95% CI −7.8–(−2.2); *p* = 0.0005), COPD (−2.2%; 95% CI −2.5–(−1.8); *p*<0.00001) as well as all hospitalisations (−1.8%; 95% CI −1.9–(−1.6); *p*<0.00001). According to the causes in German death statistics, there were only 16 notified deaths due to PNTM infection among ten males and six females between 2005 and 2011. Of those deceased, ten subjects (63%) were aged ≥65 years. Notably, none of those 16 deaths occurred inside the hospital.

## Discussion

Our study provides a detailed picture of PNTM infection-associated hospitalisations in Germany and emphasises the considerable interaction with chronic respiratory diseases, particularly COPD as the most frequent associated condition. Between 2005 and 2011, elderly men had the highest age-specific hospitalisation rate. However, while the prevalence of PNTM infection-associated hospitalisations increased significantly and regardless of the increasing overall number of hospitalisations for both sexes, it showed the most pronounced increases among females, especially those of young and middle age, and hospitalisations associated with cystic fibrosis (CF). These findings may be relevant to patient care and the allocation of resources in healthcare.

Population-based data on the epidemiology of PNTM infections in Europe are limited [11,15]. Two recent studies from France and Denmark, which analysed periods from 2001 to 2003 and from 1997 to 2008 and linked NTM isolates from respiratory specimens to population-based clinical data, found similar average annual rates of 0.7 and 1.1 PNTM infection per 100,000 population [11,15]. However, the incidence remained unchanged during the respective study periods. In this concern, our results are consistent with the upward trends of PNTM infections from other laboratory- and population-based studies around the world [8,13,16,19-21]. Our overall average annual age-adjusted hospitalisation rate of 0.9 PNTM infections per 100,000 population as well as the average age-adjusted increase of 6%/year is slightly lower, but comparable to the findings of a recent study from the United States (US) [9]. However, in line with other European studies that have shown consistently lower rates of PNTM than in North American studies [3,11,14,15], age and sex specific hospitalisation rates among elderly men and women were considerably lower in our study than in the aforementioned US study by Billinger and colleagues [9]. Furthermore, in agreement with other US studies we found that the prevalence of PNTM infection was most significantly increasing among females [3,9,13,16]. In fact, males carried the major burden of PNTM-associated hospitalisations in our study, whereas elderly women with nodular bronchiectatic disease have been considered as the predominant patient population outside Europe [3,14,16,22]. This finding supports the existence of a unique epidemiology of PNTM infections in accordance with the distinct distribution of NTM species isolated from respiratory samples within Europe and recent reports from other European countries [6,11,15,19,20,23], where PNTM infections appear to be . However, it remains to be studied whether changing host factors or a changing NTM species distribution over time account for these observations [6].

Notably, we observed a significant decline of immunodeficiencies, tuberculosis (TB), alcohol-related disorders and influenza and pneumonia among PNTM infection-associated hospitalisations. In line with previous studies observing an increase in PNTM infections, the incidence of TB and with it the number of TB-associated hospitalisations declined steadily in Germany during the study period [8,12,17,21,24,25]. However, in contrast to reports from the US and Canada [3,13,14,21], the incidence of TB may still outrange that of PNTM infections in most European countries [25].

The present study is a nationally representative population-based analysis of the burden and trends among almost 6,000 PNTM infection-associated hospitalisations, including 80% of hospitals across the country and >125 million hospitalisations over a 7-year period. However, our study has some inherent limitations. Firstly, ICD-10 diagnosis codes are primarily used for reimbursement purposes and only secondarily for epidemiological research. They may be subject to potential sources of errors, lack validation for PNTM infections and, in general, are considered to have high specificity, but only moderate sensitivity, thus being prone to an underestimation of disease prevalence [9,20,26]. Secondly, the diagnosis of PNTM infection did not require compliance with the ATS/IDSA diagnostic criteria for NTM disease [1]. Details on isolated mycobacterial species and disease presentations were unavailable. However, the fact that PNTM infection itself was the primary diagnosis in >50% of hospitalisations and severe complications like respiratory failure were frequent and significantly increasing over time suggests clinically relevant disease in the majority of hospitalisations. Thirdly, we were unable to account for readmissions, which may have had an impact on hospitalisation rates, though a substantial overestimation appears unlikely. Fourthly, several previous studies have suggested that the epidemiology of PNTM infections may be influenced by environmental, geographical and sociodemographic patterns [22,24,27,28]. However, we did not analyse the data stratified for individual federal states or urban and rural areas, mainly due to the potential bias related to regional differences of health care utilisation of medical services, and the unavailability of information on NTM species. Finally, our results apply to hospitalised populations only. PNTM infections are chronic in nature and usually require long-term follow-up care in the outpatient setting, where disease prevalence is likely different. A recent report on patient-centred care in outpatient respiratory medicine, which was based upon the billing data of 30 representative German private respiratory practices in 2010, showed that ICD-10 category A31 (infection due to other mycobacteria) accounted for on average 0.058% of outpatient diagnoses and correspondingly roughly 1,850 consultations [29]. Moreover, the fact that none of the few deaths occurred during hospitalisation, further emphasises that the major burden of disease is managed out-of-hospital. Therefore, our data are likely to underestimate the overall burden of PNTM infections. However, as data are limited, our results are the best currently available surrogate for the epidemiological trends of PNTM infections across Germany.

Altogether, the reasons for increases in PNTM cases are unknown. Increasing hospitalisation rates do not necessarily indicate increasing true incidence or prevalence of PNTM infections. They may rather be attributable to advances in laboratory culture and molecular speciation methods, increasing awareness, increasing environmental exposure, decreasing immunity to mycobacteria and/or aging and increasingly prevalent susceptible populations [4,10,12,24]. In this respect, our findings affirm the close link between PNTM infection and COPD, which has also been observed by others [11,13,14]. A recent Danish population-based case–control study demonstrated that chronic respiratory disease, particularly COPD, is a strong risk factor for PNTM disease [23]. On the other hand, a Canadian study found that the increase in pulmonary MAC disease was likely multifactorial and could not be explained exclusively by population aging, COPD and other risk factors [22]. Our finding that hospitalisation rates were not continuously increasing with age is contrary to recent studies from the US and Canada, which found the highest rates among the very advanced age group [9,13,22], and argues against more severe disease and frequent readmissions in this age group. It may rather indicate age-related differences regarding the access to health services and suggest that PNTM infections are still underdiagnosed during hospitalisations of the very advanced age group in Germany. The increasing number of unrelated comorbidities, along with the upward trend regarding the average number of secondary diagnoses per primary diagnosis of PNTM infection, may reflect the increasingly aged and comorbid population during the study period. This is supported by the fact that both the average age of hospitalised subjects and the average number of secondary diagnoses per case were steadily increasing in general for hospitalisations during the study period (from 52.5 to 54.6 years and from 3.9 to 4.8 secondary diagnoses between 2005 and 2011, respectively). Moreover, the observation that cachexia, defined as a body mass index <18.5 kg/m^2^ according to common DRG coding guidelines, was a frequent secondary condition in 11% of hospitalisations with PNTM infection as the primary diagnosis supports the existence of a distinct morphotype in a subset of patients, as recently suggested by Kim and colleagues [30]. In this study, respective subjects were taller and leaner than controls and had high rates of CF transmembrane conductance regulator (CFTR) mutations in 36%.

Our findings have several important implications. Although we were unable to estimate the costs for hospitalisations associated with PNTM infection, our data on the mean LOS confirm that PNTM infections represent an underappreciated economic healthcare burden, with considerable associated treatment costs comparable to that of other chronic infectious diseases [31]. Furthermore, the liaison between PNTM infection and chronic respiratory diseases like COPD, bronchiectasis and CF, which were all significantly increasing as associated conditions during the study period, calls for caution regarding the increasingly broad use of macrolide antibiotics as an adjunct immunomodulatory treatment [32-34]. It has been shown that macrolides block autophagy and inhibit the intracellular killing of mycobacteria [35]. As a consequence, their chronic use may hypothetically predispose to PNTM infection and, moreover, promote the emergence of macrolide resistance, which may be associated with worse outcomes [1,35]. A novel finding from our study is that young and middle-aged females showed the most pronounced increases of average age-specific hospitalisation rates of up to 17%/year. This observation may highlight the importance of genetic susceptibility, particularly CFTR mutations, and warrant extensive work-up and continued surveillance of these patients. Lastly, it is important to note that our results may not fully apply to other European countries and settings.

## Conclusions

The present study provides evidence for a unique, though changing epidemiology of PNTM infection-associated hospitalisations in Germany. However, given the rarity of PNTM infections multinational clinical research collaborations like the Non-tuberculous Mycobacteria Network European Trialsgroup (NTM-NET) are needed for future and prospective research on the epidemiology of PNTM infections across Europe and beyond.

## Abbreviations

APC: annual percentage change; ATS: American Thoracic Society; CF: Cystic fibrosis; CFTR: CF transmembrane conductance regulator; CI: Confidence intervals; COPD: Chronic obstructive pulmonary disease; DRG: Diagnosis-related groups; ICD-10: International Classification of Diseases, 10th revision; IDSA: Infectious Diseases Society of America; LOS: length of hospital stay; MAC: *Mycobacterium avium* complex; NTM: Non-tuberculous mycobacteria; PNTM: Pulmonary non-tuberculous mycobacterial; TB: Tuberculosis; US: United States.

## Competing interests

The authors declare that they have no competing interests.

## Authors’ contributions

FCR conceived and designed the study, performed the statistical analysis, interpreted the data and drafted the manuscript. RMA participated in the study design and revised the manuscript critically for important intellectual content. FCB revised the manuscript critically for important intellectual content. ADR participated in the study design and revised the manuscript critically for important intellectual content. MWP interpreted the data and contributed to the drafting of the manuscript. JR participated in the study design and revised the manuscript critically for important intellectual content. HS revised the manuscript critically for important intellectual content. DW interpreted the data and contributed to the drafting of the manuscript. TW contributed to the study design, supervised the study and revised the manuscript critically for important intellectual content. All authors read and approved the final manuscript.

## Pre-publication history

The pre-publication history for this paper can be accessed here:

http://www.biomedcentral.com/1471-2334/13/231/prepub
